# Impact of mutations on the plant-based production of recombinant SARS-CoV-2 RBDs

**DOI:** 10.3389/fpls.2023.1275228

**Published:** 2023-10-06

**Authors:** Valentina Ruocco, Ulrike Vavra, Julia König-Beihammer, Omayra C. Bolaños−Martínez, Somanath Kallolimath, Daniel Maresch, Clemens Grünwald-Gruber, Richard Strasser

**Affiliations:** ^1^ Department of Applied Genetics and Cell Biology, University of Natural Resources and Life Sciences, Vienna, Austria; ^2^ Core Facility Mass Spectrometry, University of Natural Resources and Life Sciences, Vienna, Austria

**Keywords:** antigen, glycoprotein, glycosylation, *Nicotiana benthamiana*, spike protein, vaccine, virus

## Abstract

Subunit vaccines based on recombinant viral antigens are valuable interventions to fight existing and evolving viruses and can be produced at large-scale in plant-based expression systems. The recombinant viral antigens are often derived from glycosylated envelope proteins of the virus and glycosylation plays an important role for the immunogenicity by shielding protein epitopes. The receptor-binding domain (RBD) of the SARS-CoV-2 spike is a principal target for vaccine development and has been produced in plants, but the yields of recombinant RBD variants were low and the role of the N-glycosylation in RBD from different SARS-CoV-2 variants of concern is less studied. Here, we investigated the expression and glycosylation of six different RBD variants transiently expressed in leaves of *Nicotiana benthamiana*. All of the purified RBD variants were functional in terms of receptor binding and displayed almost full N-glycan occupancy at both glycosylation sites with predominately complex N-glycans. Despite the high structural sequence conservation of the RBD variants, we detected a variation in yield which can be attributed to lower expression and differences in unintentional proteolytic processing of the C-terminal polyhistidine tag used for purification. Glycoengineering towards a human-type complex N-glycan profile with core α1,6-fucose, showed that the reactivity of the neutralizing antibody S309 differs depending on the N-glycan profile and the RBD variant.

## Introduction

The SARS-CoV-2 pandemic has dramatically shown that efforts need to be increased for the fast and efficient development of counter measurements to combat newly emerging viruses. The rapid production of potent vaccines is one way to fight emerging and reemerging viral pathogens. The heavily glycosylated SARS-CoV-2 spike protein that is exposed at the viral surface is one of the key targets for vaccine development. Glycosylation is a major posttranslational modification that is found on a vast number of mammalian proteins and on surface proteins of viruses that use mammalian cells as a host. Since glycosylation is important for virus transmission and cell entry, understanding of biological processes governed by glycosylation is crucial to prevent virus infection and disease progression. N-glycosylation is the major type of glycosylation found on SARS-CoV-2 and other enveloped viruses (Watanabe et al., 2020b). N-glycosylation is initiated in the endoplasmic reticulum (ER) by transfer of a preassembled oligosaccharide to specific asparagine residues present on nascent polypeptide chains (Strasser, 2016). While the attached N-glycans promote protein folding and serve important functions during quality control in the ER, the complex type N-glycans that are generated in the Golgi apparatus have more diverse functions. For example, a single fucose residue is transferred in the Golgi apparatus to the conserved complex N-glycan present in the IgG1 heavy chain. The presence of this core fucose alters the affinity of antibodies to cellular receptors and thus controls effector functions (Wang and Ravetch, 2019). Other complex N-glycan modifications on viral or recombinant glycoproteins enable the interaction with specific lectin-type receptors (Hoffmann et al., 2021).

The SARS-CoV-2 spike protein monomer on the surface of the virus contains 22 highly conserved N-glycosylation sites. N-glycosylation of these sites is important for expression, conformational dynamics and virus infectivity as shown for the original SARS-CoV-2 strain (Yang et al., 2020b; Zhao et al., 2020; Newby et al., 2023). Many viral envelope proteins and subunits are explored as vaccine candidates and glycosylation is critical for vaccine development (Bagdonaite and Wandall, 2018; Ozdilek and Avci, 2022). Glycosylation is frequently required for proper folding of viral antigens expressed in heterologous systems (Casalino et al., 2020). The attached N-glycans are bound by lectin-like molecular chaperones calnexin/calreticulin which promote folding (Shin et al., 2021; Margolin et al., 2023). Moreover, the presence or absence of glycans on vaccines can have completely detrimental effects on the potency. For example, masking of epitopes by attachment of additional glycans can divert the immune response to distinct regions of the polypeptide and enhance the induction of neutralizing antibodies (Shi et al., 2022). Deletion of glycans can also be beneficial and enhance the immune response with a broader protection as shown for SARS-CoV-2 vaccination approaches (Huang et al., 2022; Wu et al., 2022). In addition to glycan occupancy of viral proteins, also the glycan structure has an impact on the immunogenicity as indicated by differences in reactivity with specific SARS-CoV-2 neutralizing antibodies (Pinto et al., 2020; Samuelsson et al., 2022). Since its emergence, SARS-CoV-2 has undergone continuous mutations and recent evidence shows that some variants of concern like Delta or Omicron display differences in N-glycan processing (Zheng et al., 2022; Baboo et al., 2023) which may affect the infectivity like shown for the Omicron BA.1 variant (Lusvarghi et al., 2023). These findings underscore the importance of N-glycosylation for SARS-CoV-2 virus infection (Gong et al., 2021; Maity and Acharya, 2023) and the requirement for platforms and expression systems that allow the production of defined homogenous glycan structures on recombinant viral proteins (Yang et al., 2015; Schwestka et al., 2021; Hsu et al., 2023). Since even small differences in glycosylation may alter the immune response (Samuelsson et al., 2022), a controlled glycosylation profile on protein subunit vaccines is highly desirable.

The receptor-binding domain (RBD) of the spike protein binds to the human angiotensin-converting enzyme 2 (ACE2) receptor when RBD is exposed in the up state. RBD has two N-glycans at sites N331 and N343 that are preserved in all SARS-CoV-2 variants of concern. The N343 glycan plays an important role in shielding RBD in the down state and through glycan-protein interaction it contributes to opening of the spike protein for receptor binding (Sztain et al., 2021; Pang et al., 2022). The N331 glycan is involved in the interaction of the spike protein with the glycocalyx of the host cell and thus also contributes to the host cell infectivity (Mycroft-West et al., 2020; Kim et al., 2022). RBD carries dominant neutralizing epitopes and is therefore a prime candidate for protein subunit vaccines (Yang et al., 2020a).

In recent years, plants have emerged as commercially relevant production systems for recombinant biopharmaceuticals like antibodies or vaccines (Chung et al., 2022; Eidenberger et al., 2023). Transient expression in plants provides a very flexible platform for safe and fast production of recombinant proteins. Using transient production in *Nicotiana benthamiana* a virus-like particle vaccine (Covifenz®) was produced by the company Medicago and approved by Health Canada as COVID-19 vaccine (Hager et al., 2022). Despite the fact that production of Covifenz® was not continued and Medicago ceased operations due to a management decision from its parent company (Benvenuto et al., 2023), the approval showed that this plant-made vaccine is safe and effective in preventing COVID-19. An RBD-based subunit vaccine that was effective, safe and non-toxic in animal studies is currently tested in phase 1 clinical trials (Phoolcharoen et al., 2023). In several other studies the huge potential of transient expression in plants for RBD subunit vaccine production has been demonstrated (Ruocco and Strasser, 2022). Most of these studies focused on the original RBD sequence. By contrast, comparable little information is available how mutations in different variants affect the plant-based production and glycosylation of recombinant RBD. Here, we produced glycoengineered RBD from different SARS-CoV-2 variants and functionally characterized the plant-produced viral proteins to reveal challenges associated with their production in *N. benthamiana*.

## Results

### The yield of transiently expressed RBD-215 variants differs

**Figure 1:**
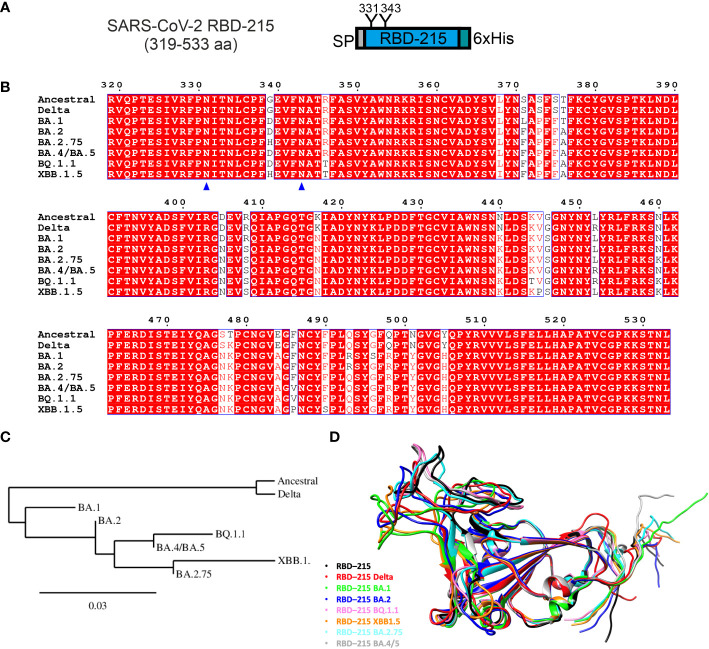
Comparison of expressed RBD-215 variants. **(A)** Schematic illustration of the expressed SARS-CoV-2 RBD-215 protein. SP, signal peptide; “Y”, N-glycosylation sites, numbering according to the full-length SARS-CoV-2 spike sequence (UniProt: P0DTC2); 6xHis, polyhistidine tag composed of six histidine residues. **(B)** Sequence alignment of the amino acid region 319-533 from different SARS-CoV-2 variants was done using T-Coffee (https://www.ebi.ac.uk/Tools/msa/tcoffee/) and displayed using ESPript3.0 (https://espript.ibcp.fr/ESPript/cgi-bin/ESPript.cgi). N-glycosylation sites are marked by blue triangles. **(C)** Phylogenetic tree of RBD-215 sequences constructed using Phylogeny.fr (https://www.phylogeny.fr/). **(D)** Comparison of RBD-215 models produced by AlphaFold CoLab, showcasing their distinct and common structures and secondary features. The models were superimposed using the MatchMaker tool from UCSF Chimera (https://www.cgl.ucsf.edu/chimera/), which allows a direct comparison, highlighting conserved regions and structural variations (see also **Table S1**).

In previous studies, we have transiently expressed recombinant SARS-CoV-2 RBD (Wuhan-Hu-1 strain, region R319-L533 from the spike protein, length 215 amino acids and here referred to as RBD-215) by agroinfiltration in *N. benthamiana* leaves (Schwestka et al., 2021; Shin et al., 2021; König-Beihammer et al., 2022). The expressed protein has the barley α-amylase signal peptide for targeting the protein to the secretory pathway, two N-glycosylation sites (N331 and N343, numbering according to the full-length SARS-CoV-2 spike sequence) (Watanabe et al., 2020a) and a polyhistidine-tag attached to the C-terminus (**Figure 1A**). We used the same expression vector (pEAQ-*HT*) (Sainsbury et al., 2009) to examine the effect of different mutations occurring in the RBD of SARS-CoV-2 variants on their expression and functionality (**Figure 1B**). We aimed to express seven RBD-215 variants (Delta, Omicron sublineages BA.1, BA.2, BA.2.75, BA.4/BA.5, BQ.1.1 and XBB.1.5) (**Figures 1C, D**; **Table S1**). DNA sequences coding for the RBD-215 variants were codon-optimized for *N. benthamiana* and *in vitro* synthesized. Despite several attempts, no positive transformation clones were obtained for the BA.4/BA.5 sequence cloned into the expression vector pEAQ-*HT* and this variant was therefore not further investigated. The remaining six RBD-215 variants were all successfully cloned into pEAQ-*HT*.

**Figure 2:**
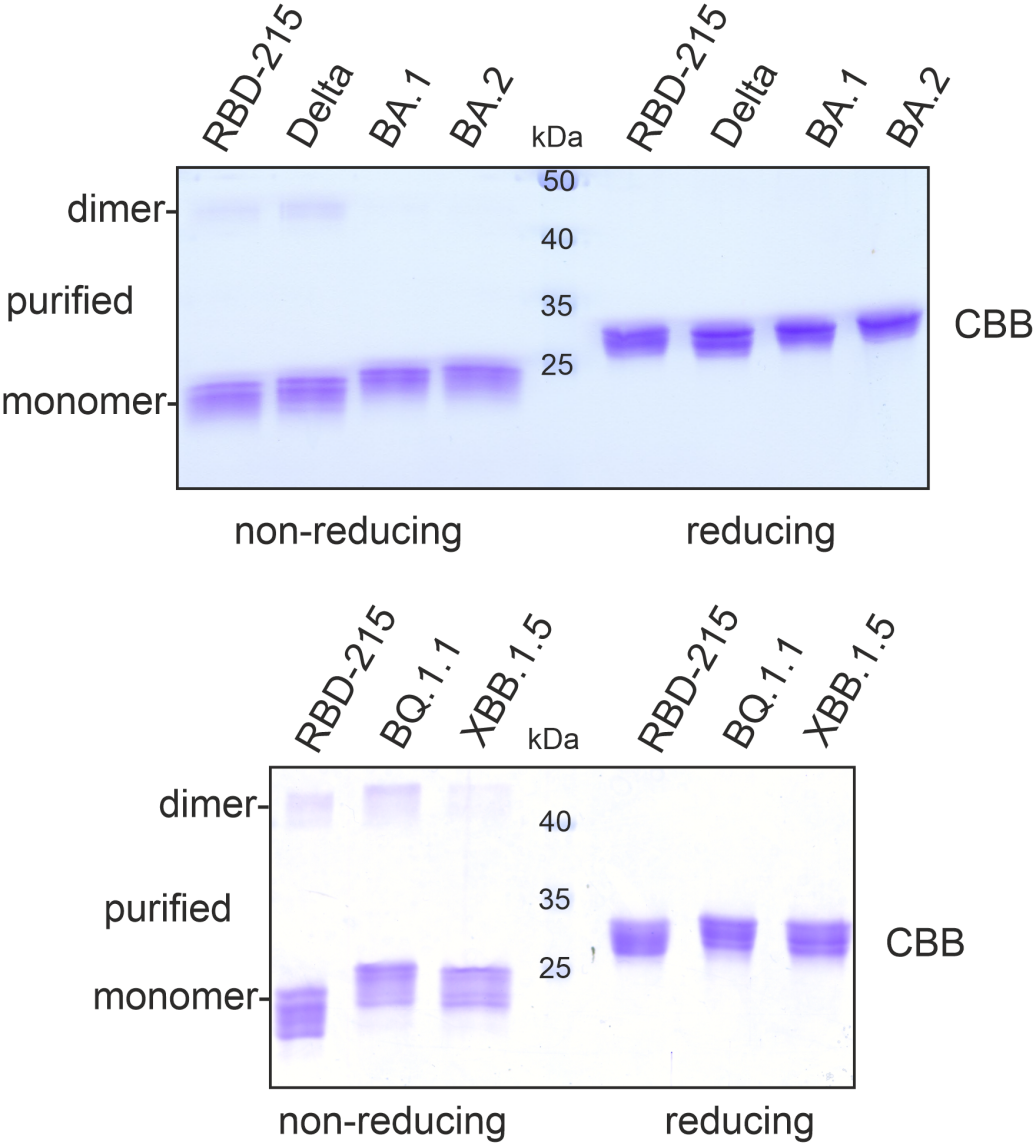
SDS-PAGE of purified RBD-215 variants. IMAC-purified RBD-215 variants were subjected to SDS-PAGE under non-reducing and reducing conditions and stained with Coomassie Brilliant Blue (CBB).

To compare the yield of the transiently expressed RBD-215 variants, *Agrobacteria* carrying the respective pEAQ-*HT* expression constructs were infiltrated into leaves of *N. benthamiana* ΔXT/FT plants which are glycoengineered to produce mainly complex N-glycans lacking β1,2-xylose and core α1,3-fucose (Strasser et al., 2008). 4 days after infiltration, leaves were harvested and polyhistidine-tagged RBD-215 variants were purified from the apoplastic fluid via immobilized metal affinity chromatography (IMAC). All variants except BA.2.75 could be purified and displayed mainly the monomeric RBD-215 form upon SDS-PAGE under non-reducing conditions which is consistent with previous data (**Figure 2**) (Shin et al., 2021). For some variants an additional faint band between 40 and 50 kDa was visible under non-reducing conditions which disappeared under reducing conditions. This band likely comes from a dimer formed by intermolecular disulfide bonds.

The purification yield of the Delta variant ranged from 10 – 20 µg/g fresh leaves and was comparable to yields for RBD-215 (10-20 µg/g fresh leaves; Shin et al., 2021). For Omicron variants BA.1 and BA.2, 5-10 µg/g fresh leaves were obtained by purification. By contrast, only small amounts could be purified from BQ.1.1 (1-2 µg/g fresh leaves) and XBB.1.5 (~ 0.5 µg/g fresh leaves).

### The RBD-215 variants display differences in stability in the apoplastic fluid

**Figure 3:**
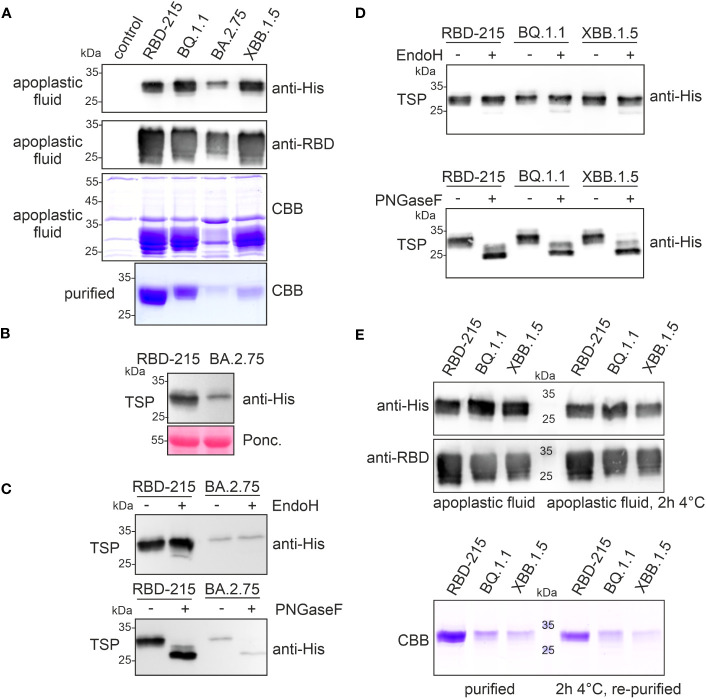
RBD-215 variants display differences in expression and yield after purification. **(A)** Immunoblot analysis (anti-histidine and anti-RBD antibodies) and SDS-PAGE with CBB-staining of isolated apoplastic fluid and purified RBD-215 variants. **(B)** Immunoblot of total soluble protein (TSP). Leaves from infiltrated *N. benthamiana* ΔXT/FT plants were harvested 4 days after infiltration and TSP was analysed with anti-histidine antibody. **(C, D)** Immunoblot of Endo H or PNGase F digested TSP. TSP was obtained by infiltration of *N. benthamiana* ΔXT/FT with *Agrobacteria* carrying the expression vectors for the indicated RBD-215 proteins. **(E)** Immunoblot and SDS-PAGE with CBB staining of the apoplastic fluid. The apoplastic fluid or the purified proteins were incubated for 2 h at 4°C followed by SDS-PAGE and immunoblotting or IMAC-purification (re-purified) and SDS-PAGE with CBB staining.

The yield of BQ.1.1 and XBB.1.5 was 10-20 times lower and even further reduced for BA.2.75. Therefore, we investigated the expression and fate of these three low-yield variants more in detail and included RBD-215 for comparison. When we analysed the apoplastic fluid, the signal intensity was comparable for RBD-215, BQ.1.1 and XBB.1.5, but the amount of polyhistidine-tagged BA.2.75 was much lower (**Figure 3A**). Consistent with the immunoblot analysis, the apoplastic fluid isolated from BA.2.75 expressing plants displayed less RBD-215-specific bands and the amount of BA.2.75 was also reduced in total soluble protein showing that the expression of this variant is lower (**Figure 3B**). Previously we found that poorly expressing RBD variants are misfolded and retained in the ER (Shin et al., 2021). ER-retained proteins typically carry Endo H-sensitive oligomannosidic N-glycans. BA.2.75 in total soluble protein was resistant to Endo H, but the N-glycans could be cleaved by PNGase F which can remove oligomannosidic and Golgi-processed complex N-glycans lacking core α1,3-fucose (**Figure 3C**). Likewise, BQ.1.1 and XBB.1.5 present in total soluble protein extracts exhibited mainly Endo H-resistant glycans (**Figure 3D**). Taken together, these findings indicate that the expressed RBD-215 variants are not misfolded and secreted through the Golgi to the apoplast.

Immunoblots and SDS-PAGE of proteins isolated from the apoplastic fluid confirmed that the expression levels of RBD-215, BQ.1.1 and XBB.1.5 are comparable (**Figures 3A, E**). Therefore, in contrast to BA.2.75, the low yield of XBB.1.5 cannot be explained by lower expression levels or an inaccessible tag. On Coomassie Brilliant Blue-stained gels, RBD-215, BQ.1.1 and XBB.1.5 exhibited different faster migrating bands that likely represent RBD-215 protein with a cleaved polyhistidine-tag because these proteins are not detectable on immunoblots. It is therefore conceivable that the RBD-215 variants differ in their susceptibility to cleavage of the polyhistidine-tag which impacts their purification and overall yield. Indeed, the RBD-215 variants lost their polyhistidine tag in the apoplastic fluid rather rapidly, and this instability correlates with the observed low yields during IMAC purification (**Figure 3E**). A faster IMAC purification protocol with magnetic beads and continuous cooling resulted in a more than threefold (~1.8 µg/g fresh weight) increase of the yield for XBB.1.5. However, even under these conditions, the polyhistidine tag on XBB.1.5 was more unstable compared to RBD-215 or BQ.1.1, as less protein was purified after incubation for 2h at 4°C (**Figure 3E**).

### The RBD-215 variants are glycosylated and functional

**Figure 4:**
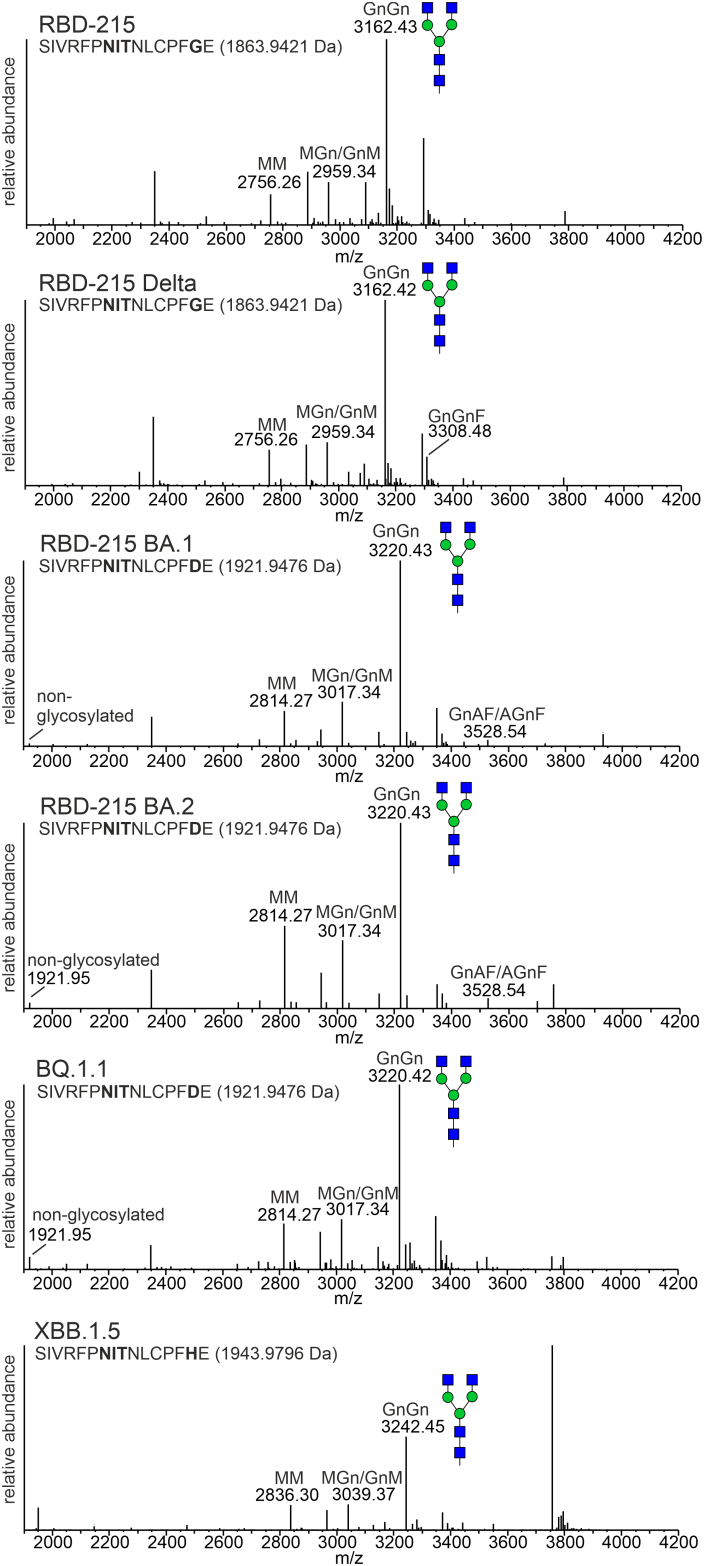
MS spectra of the RBD-215 glycopeptide containing N-glycosylation site N331 of the SARS-CoV-2 spike protein. The assigned N-glycan structures were labelled according to the ProGlycAn nomenclature (http://www.proglycan.com/). A cartoon illustration (filled green circle, mannose; filled blue square, GlcNAc; for details see http://www.functionalglycomics.org/) highlights the main N-glycan structures detected for each peptide.

Next, we analysed the N-glycans to see if the mutations affect the N-glycan occupancy and/or N-glycan processing. Purified RBD-215 proteins were proteolytically digested and glycopeptides were analysed by mass spectrometry (MS). All purified RBD-215 variants carried primarily complex N-glycans with GlcNAc_2_Man_3_GlcNAc_2_ (GnGn) as major N-glycan on both N-glycosylation sites (**Figure 4**; **Supplemental Figure S1**). In addition, all RBD-215 variants displayed small amounts of truncated N-glycans with removed terminal GlcNAc residues (MM and GnM N-glycans). These N-glycans are likely generated in the apoplast by β-hexosaminidase 3 (HEXO3) (Shin et al., 2017). On Omicron BA.2 the amounts of truncated N-glycans were modestly increased on both N-glycosylation sites suggesting that the N-glycans are more accessible for removal of terminal GlcNAc residues (**Table 1**). The overall very low amounts of oligomannosidic N-glycans (0.0-3.4%) are consistent with the secretion trough the Golgi and high accessibility of the N-glycans for processing. In all variants more than 96% of both N-glycosylation sites are fully glycosylated showing that both sites are very well recognized by the plant oligosaccharyltransferase complex (Castilho et al., 2018).

**Table 1:** Relative N-glycan composition at the two N-glycosylation sites of the RBD-215 variants.

Site N331	RBD-215^5^	Delta^5^	BA.1^6^	BA.2^6^	BQ.1.1^6^	XBB.1.5^7^
**Peptide^1^**	1,6	2,5	1,0	1,4	3,4	1,5
**Truncated^2^**	16,3	14,0	13,0	23,2	17,1	16,8
**Complex^3^**	82,1	83,4	85,2	73,5	78,9	80,7
**Mannosidic^4^**	0,0	0,1	0,8	1,9	0,6	1,0
**Total**	100	100	100	100	100	100

Site N343	RBD-215^8^	Delta^8^	BA.1^8^	BA.2^8^	BQ.1.1^9^	XBB.1.5^9^
**Peptide^1^**	0,1	0,1	0,1	0,1	0,0	0,0
**Truncated^2^**	15,9	13,9	12,5	21,7	20,6	26,8
**Complex^3^**	83,4	85,5	85,4	74,8	78,4	71,3
**Mannosidic^4^**	0,6	0,5	2,1	3,4	1,0	1,9
**Total**	100	100	100	100	100	100

^1^non-glycosylated peptide. ^2^processed N-glycans lacking GlcNAc residues at the non-reducing end (MM, MU, MMF, MUF) or carrying only Asn-GlcNAc. ^3^complex N-glycans with at least one GlcNAc residues at the non-reducing end (e.g. GnGn, MGn/GnM, GnA/AGn, GnGnF, MGnF/GnMF, GnAF/AGnF). ^4^mannosidic N-glycans ranging from Glc1Man9 to Man4. ^5^peptide: SIVRFP**NIT**NLCPFGE; ^6^peptide: SIVRFP**NIT**NLCPFDE; ^7^peptide: SIVRFP**NIT**NLCPFHE; ^8^peptide: VF**NAT**RFASVYAWNRK; ^9^peptide: VF**NAT**TFASVYAWNRK.

**Figure 5:**
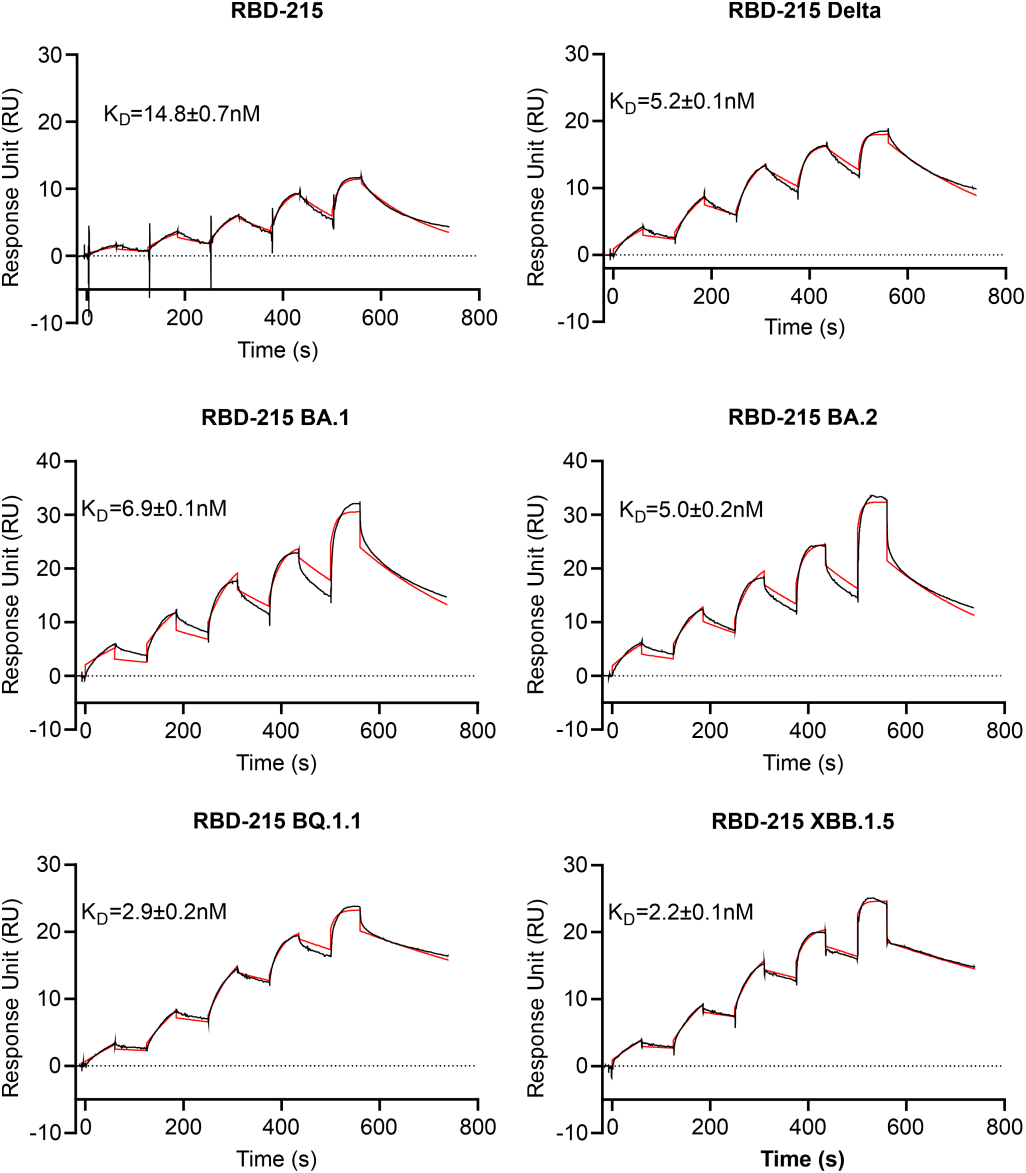
ACE2-Fc receptor binding of RBD-215 variants. SPR sensorgrams are shown and the K_D_ values (mean ± SD, n = 3) are given (red line fitted curves).

To examine whether the RBD-215 variants are functional in terms of binding to the human cellular receptor ACE2, we carried out binding assays using SPR. All six RBD-215 variants displayed binding to ACE2-Fc (**Figure 5**; **Table S2**). The binding curves for some variants appeared slightly out of phase which can be attributed to protein-intrinsic properties. By contrast, a comparison of SEC and non-SEC purified RBD-215 and BA.1 showed that the small amounts of dimers or oligomers do not affect the binding (**Supplemental Figure S2**). The binding affinity of the more recently evolved variants BQ.1.1. and XBB.1.5 was higher than RBD-215, Delta, BA.1 and BA.2 being consistent with the evolution of the virus towards better binding to ACE2 and higher infection capability (Ito et al., 2023; Yue et al., 2023).

**Figure 6:**
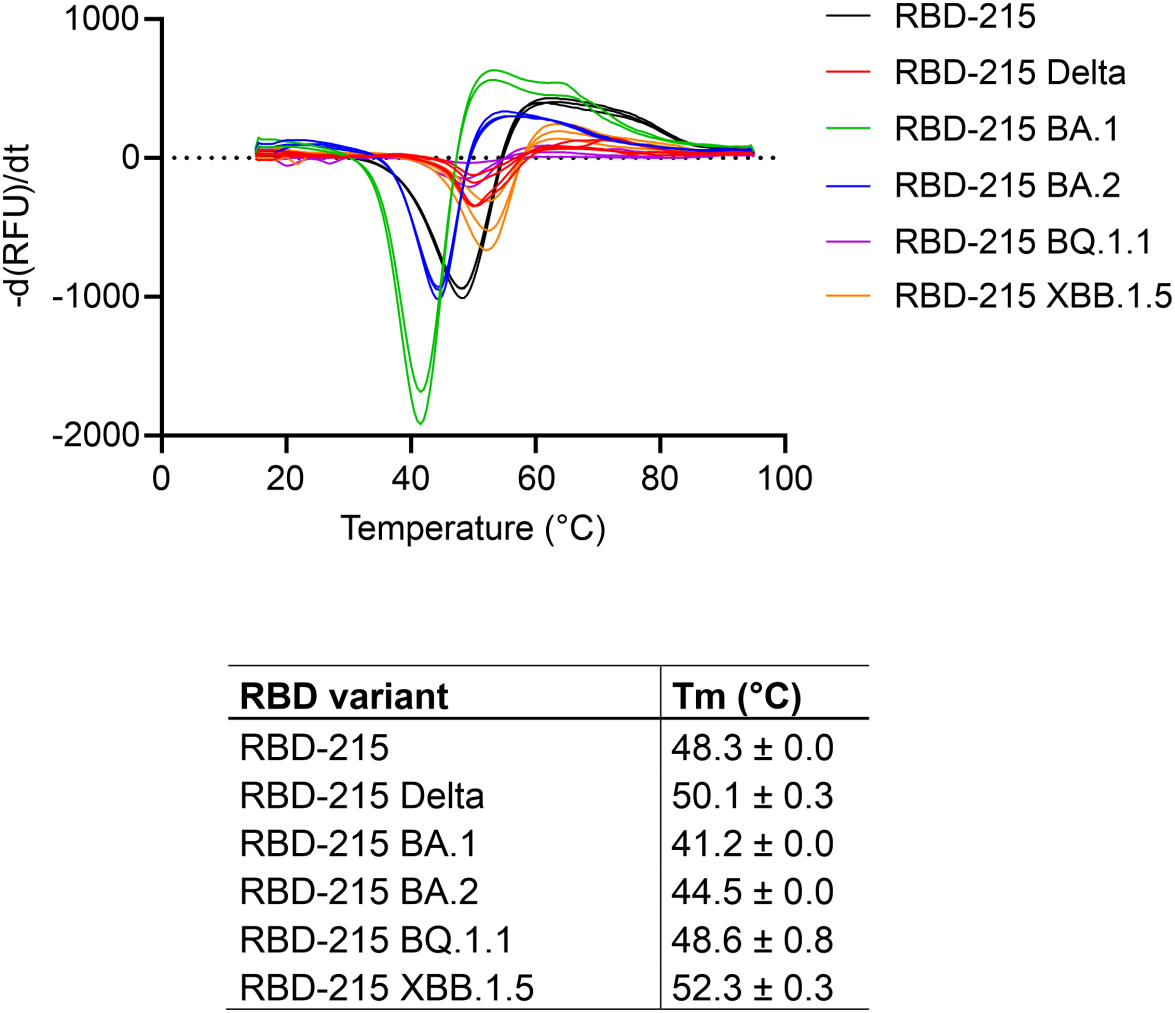
Thermal stability of RBD-215 variants. DSF profiles of the RBD-215 variants show changes in protein intrinsic fluorescence. For each RBD-215 protein, three overlaid curves (technical replicates) are shown and the Tm is given for every protein (mean ± SD, n = 3).

To further investigate characteristics of the plant-produced RBD-215 variants, we analysed the thermostability of the purified proteins using differential scanning fluorimetry (DSF). The RBD-215 and Delta variants displayed a higher melting temperature (48.3°C and 50.1°C, respectively) than the Omicron variants BA.1 (41.2°C, lowest melting temperature of the analysed variants) and BA.2 (44.5°C). The reduced stability of BA.1 compared to BA.2 and the other variants is in close agreement with thermostability assays using mammalian-cell produced RBDs (Lin et al., 2022; Stalls et al., 2022) suggesting that the plant-produced variants display a similar folding. The thermostability of RBD-215 BQ.1.1 was 48.6°C and XBB.1.5. showed an increased (52.3°C) melting temperature (**Figure 6**; **Supplemental Figure S3**) which is consistent with the presence of amino acid changes (e.g. F486P) that are predicted to increase the stability (Verkhivker et al., 2023).

### Glycoengineering shows improved binding of S309 to fucosylated RBD-215 Omicron BA.1

**Figure 7:**
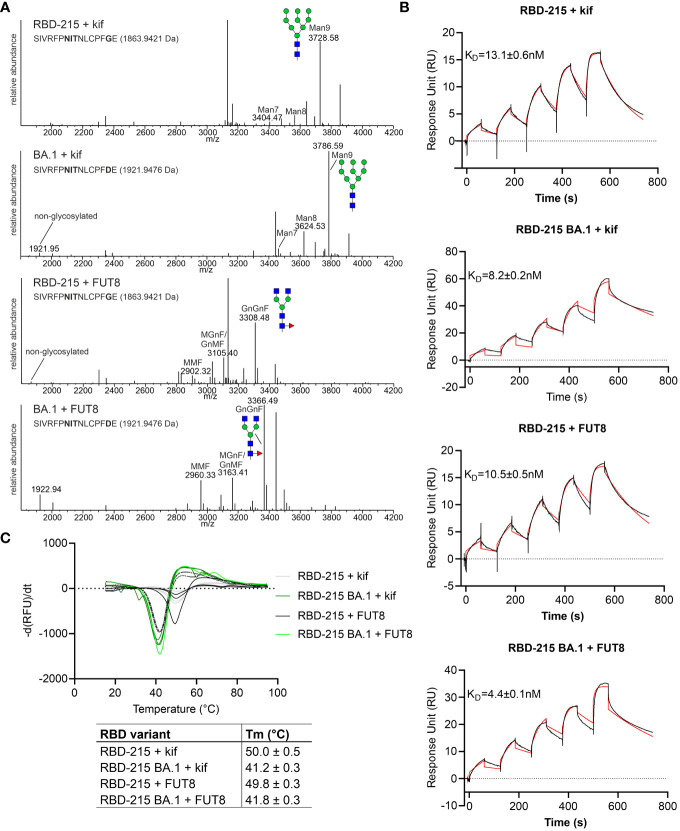
Glycoengineering results in RBD-215 variants with oligomannosidic or fucosylated complex N-glycans whose Tm and K_D_ values are not affected by changes in N-glycan structures. **(A)** MS spectra of RBD-215 + kif, Omicron BA.1 + kif, RBD-215 + FUT8, Omicron BA.1 + FUT8. A cartoon illustration (filled green circle, mannose; filled blue square, GlcNAc; red triangle, fucose) highlights the main N-glycan structures detected for each peptide **(B)** SPR analysis of ACE2-Fc binding to RBD-215 variants and **(C)** DSF curves and values (mean ± SD, n = 3) of RBD-215 + kif, Omicron BA.1 + kif, RBD-215 + FUT8, Omicron BA.1 + FUT8.

Distinct glycans attached to viral glycoproteins used as vaccine candidates may alter the immune response (Lusvarghi et al., 2023; Margolin et al., 2023). It is therefore of interest to produce glycosylation variants by glycoengineering to make vaccine candidates more potent. MS analysis of the N-glycans attached to N331 and N343 did not reveal huge differences in N-glycan composition (**Figure 4**). To see if RBD-215 variants can be subjected to glycoengineering in plants, we expressed RBD-215 and Omicron BA.1 in the presence of kifunensine to block α-mannosidases and produce oligomannosidic N-glycans. In another approach, we co-expressed the human core α1,6-fucosyltransferase (FUT8) to generate complex N-glycans carrying core α1,6-fucose as the vast majority of the complex N-glycans from mammalian cell-produced spike and RBD carry core fucose (Allen et al., 2021; Hsu et al., 2023). MS analysis showed that oligomannosidic N-glycans were present when kifunensine was co-infiltrated (**Figure 7A**; **Supplemental Figure S4**; **Table 2**). Upon FUT8 expression, complex N-glycans of RBD-215 and Omicron BA.1 were efficiently modified with fucose residues (~76-87% of all N-glycans fucosylated, **Table 2**). To examine the impact of the glycoengineered variants on receptor binding and thermostability we carried out SPR analysis and DSF. The K_D_ values were almost identical to the GnGn containing glycoforms (**Figures 5**, **7B**) and the melting points were in the same range (see **Figure 6**) suggesting that folding and the thermostability of the RBD-215 variants are not affected by changes in N-glycan composition (**Figure 7C**).

**Table 2:** Relative N-glycan composition of glycoengineered RBD-215 and Omicron BA.1.

Site N331	RBD-215 + FUT8	BA.1 + FUT8	RBD-215 + kif	BA.1 + kif
**Peptide**	1,7	0,2	3,0	2,4
**Truncated**	6,9	14,2	1,8	1,6
**Complex**	87,6	81,4	0,0	0,0
**Mannosidic**	3,8	4,2	95,2	96,0
**Total**	100	100	100	100
**Fucosylated**	76,7	82,3	0,0	0,0

Site N343	RBD-215 + FUT8	BA.1 + FUT8	RBD-215 + kif	BA.1 + kif
**Peptide**	0,0	0,0	0,1	0,1
**Truncated**	10,4	11,7	1,6	0,8
**Complex**	87,7	84,4	0,0	0,0
**Mannosidic**	1,9	3,9	98,3	99,1
**Total**	100	100	100	100
**Fucosylated**	86,5	86,0	0,0	0,0

**Figure 8:**
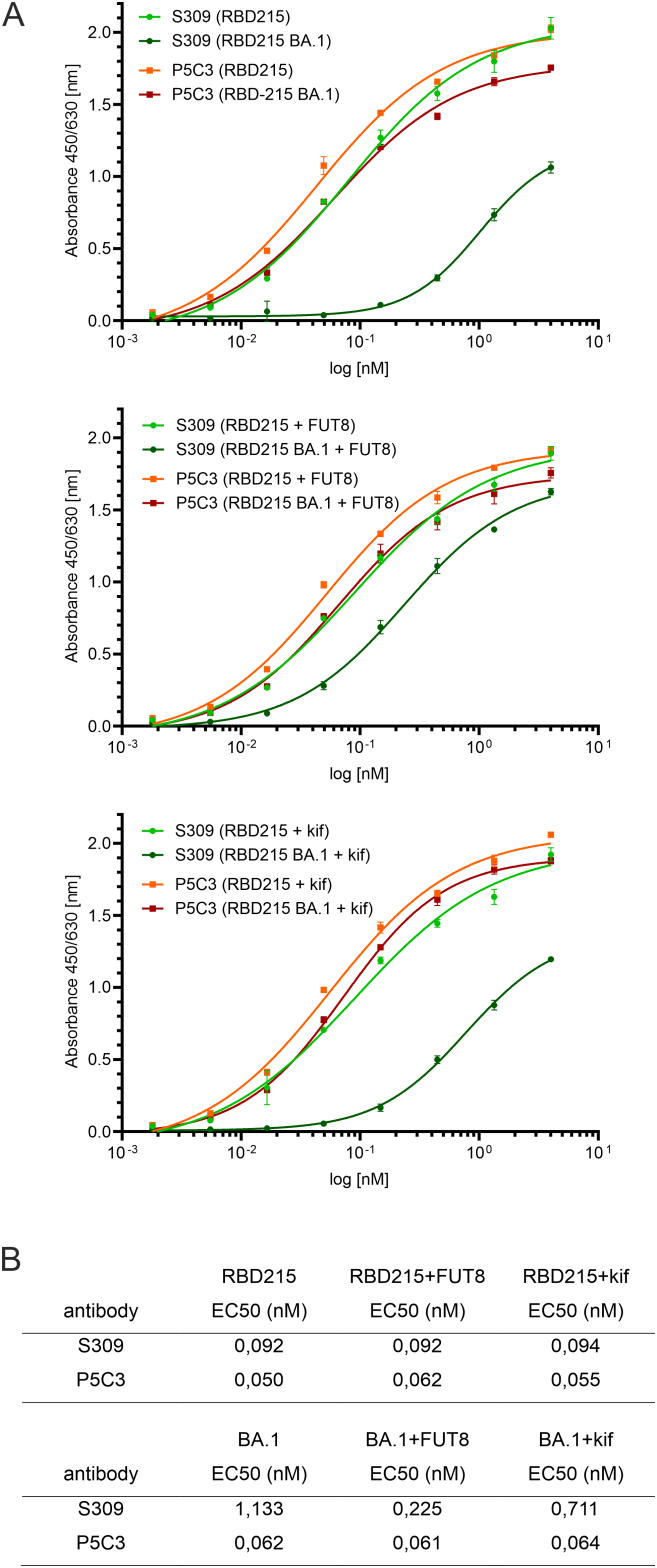
ELISA showing differences in binding of S309 antibody to glycoengineered Omicron BA.1. Binding to antibody P5C3 was used for comparison. **(A)** ELISA binding curves. **(B)** EC50 values (mean values, n = 3).

Next, we examined whether two well-characterized SARS-CoV-2 neutralizing antibodies (S309 and P5C3) (Pinto et al., 2020; Fenwick et al., 2021) can bind to RBD-215 and Omicron BA.1 as well as glycovariants thereof. P5C3 is a class 1 antibody that binds to the receptor binding motif of RBD in the “up” conformation (Fenwick et al., 2022; Chen et al., 2023). The monoclonal class 3 antibody S309 whose binding site does not overlap with the receptor binding motif, binds to an epitope that includes the fucose attached to the N-glycan on site N343 (Pinto et al., 2020; Qu et al., 2023). S309 and P5C3 bound quite similar to RBD-215 and neither the presence of fucose (RBD-215 + FUT8) nor oligomannosidic N-glycans (RBD-215 + kif) affected their binding (**Figures 8A, B**). In contrast to P5C3, S309 binding to Omicron BA.1 was strongly affected, but the reduced binding was improved 5-fold in the presence of the core fucose (BA.1 + FUT8) (**Figure 8B**). RBD-215 BA.1 with oligomannosidic N-glycans (BA.1 + kif) exhibited a modest (~1.6-fold) increase in binding to S309. These findings indicate that the fucosylated N-glycan at N343 plays a more important role for binding of the class 3 neutralizing antibody S309 to the Omicron BA.1 variant. In summary our data demonstrate that plant-produced RBD-215 variants are functional and behave like mammalian cell-derived RBD antigens.

## Discussion

Viral glycoprotein antigens produced in different expression systems are used for vaccination and can elicit protecting neutralising antibodies (Yang et al., 2020a; Allen et al., 2021). However, glycosylation is species- and cell-type specific and the production of more authentic glycosylation profiles or modification with distinct sugar residues can prevent a skewed immune response and lead to more efficient immunogens. Challenges in RBD protein production have been observed in different expression systems (Chen et al., 2021; Guo et al., 2021; Dalvie et al., 2022) including plants which displayed generally low yields (Diego-Martin et al., 2020; Shin et al., 2021; Siriwattananon et al., 2021). Here, we found differences in the yield after purification between the variants. For some RBD-215 variants the low yield is not a primary consequence of low expression levels, but rather related to proteolytic processing of the polyhistidine-tag. Degradation or cleavage of tags is a common limitation of plant-produced secreted recombinant proteins (Gattinger et al., 2021). Optimization of production requires either a protein design that reduces the susceptibility to tag cleavage or identification and inactivation of the involved proteases in the apoplastic fluid (Jutras et al., 2020). An alternative strategy is the attachment of a KDEL ER-retrieval motif that results in the accumulation of recombinant proteins in the ER and therefore prevents the contact with proteases present in the apoplast. The drawbacks of this strategy are, however, the modification of recombinant proteins with an additional peptide motif (KDEL or SEKDEL) that could result in an unwanted immune response (Petruccelli et al., 2006), inefficient purification of polyhistidine-tagged proteins from total soluble protein extracted from plant tissues, and ER inherent oligomannosidic N-glycans that interfere with glycoengineering approaches and could affect the viral antigen function.

In the analysed plant-produced RBD variants, the two N-glycosylation sites, N331 and N343, are almost fully occupied with N-glycans which has also been shown for mammalian cell-produced RBD (Allen et al., 2021; Hsu et al., 2023). In addition, we detected only minor differences in the N-glycan profile of the variants and found mainly complex N-glycans that are the dominant glycan structures on mammalian cell-produced RBD, on recombinant spike trimers, virus derived spike trimer and a plant-produced virus-like particle (Allen et al., 2021; Brun et al., 2021; Gstöttner et al., 2021; Balieu et al., 2022; Hsu et al., 2023). In contrast to that, the N-glycan profile of plant-produced trimeric HexaPro spike differs markedly from the HEK293-produced one (Margolin et al., 2023). The plant HexaPro spike displayed predominately unprocessed oligomannosidic N-glycans. While the plant HexaPro spike elicited neutralizing antibodies in hamsters, the titers were lower compared to hamsters immunized with HEK293 HexaPro spike and animals were less well protected against the virus. This highlights that the differences in N-glycan composition have an impact on the potency of the produced SARS-CoV-2 vaccines and binding of neutralizing antibodies (Lusvarghi et al., 2023).

In a previous study, no differences were found in the binding affinity between RBD carrying different types of N-glycans and S309 antibody (Hsu et al., 2023). The experiments were carried out with the original RBD sequence and are consistent with our ELISA data. By contrast, we observed a clear fucose-dependent binding of S309 to the Omicron BA.1 variant. Due to mutations in the S309 epitope which includes G339D close to N343, BA.1 binds less well to S309 (Dejnirattisai et al., 2022; Qu et al., 2023). Our data show that the presence of the core fucose in the S309 epitope can to some extent compensate for the reduced binding to BA.1. For newly emerging variants, recombinant subunit vaccines with discrete glycoforms should therefore be explored to improve the immune response.

In summary, the characteristics of the produced RBD-215 variants were very similar to mammalian cell-produced RBD which emphasizes that the transient plant-based expression system is highly suitable for RBD subunit vaccine production. Our findings are relevant for attempts aiming at the production of mosaic or cocktail vaccines in plants to induce an immune response that protects against a wide range of SARS-CoV-2 variants (Cohen et al., 2022; Zhang et al., 2022). In a previous study, a Delta RBD-Fc fusion was produced in *N. benthamiana* and shown to elicit broadly neutralizing antibodies against different strains in cynomolgus monkeys (Khorattanakulchai et al., 2022). A combination of plant-produced RBD variants in one vaccine could result in an even broader protection against circulating and emerging variants. The control of the N-glycan structures on plant-produced viral antigens could further improve such vaccines. The production of potent vaccines in plants will allow a more cost-effective manufacturing which is very important for low- and middle-income countries and thus contributes to overcome current inequities in access to such biologicals.

## Materials and methods

### Cloning of RBD-215 expression constructs

The cloning of pEAQ-RBD-215 carrying the original SARS-CoV-2 WT was described previously (Shin et al., 2021). The RBD-215 variants were cloned in the same way. Briefly, *N. benthamiana* codon-optimized DNA fragments harbouring the coding sequence for the barley α-amylase signal peptide, the RBD of SARS-CoV-2 (amino acids 319-533) and a 6x-histidine tag were PCR amplified using flanking primers, *Age*I/*Xho*I digested and ligated into *Age*I/*Xho*I digested plant expression vector pEAQ-*HT* (Sainsbury et al., 2009). The pEAQ-*HT* plant expression vectors containing RBD-215 sequence variants were transformed into *Agrobacterium tumefaciens* strain UIA143 (Strasser et al., 2005).

### Purification of his-tagged RBD-215 variants

5-week-old *N. benthamiana* ΔXT/FT plants (Strasser et al., 2008) grown at 24°C were used for transient expression of RBD-215 variants. Leaves were manually infiltrated with *Agrobacteria* (OD_600 _= 0.2) carrying the respective pEAQ-RBD-215 expression vector. For the purification, infiltrated leaves were harvested 4 days after infiltration and intracellular fluid was collected by low-speed centrifugation as described in detail previously (Castilho et al., 2011b). His-tagged RBD-215 variants were purified from collected intracellular fluid by loading onto a 5 ml HisTrap HP column (Cytiva), elution with imidazole and subsequent dialysis and concentration by ultracentrifugation as described in detail previously (Göritzer et al., 2019). For production of RBD-215 variants with oligomannosidic N-glycans, 50 µM kifunensine (Santa Cruz Biotechnology) was co-infiltrated with the *Agrobacteria* suspension. For production of RBD-215 variants with core α1,6-fucose on complex N-glycans, a previously described FUT8 expression vector was used (Castilho et al., 2011a) and *Agrobacteria* harboring the FUT8 expression vector were co-infiltrated with an OD_600 _= 0.1.

For faster purification, the intracellular fluid in 20 mM Na_2_HPO_4_, 500 mM NaCl, 10 mM imidazole, pH 7.4 was incubated with pre-washed His Mag Sepharose® Ni magnetic beads (Cytiva) and incubated at 4°C for 30 min. After removal of the magnetic particles and washing with 20 mM Na_2_HPO_4_, 500 mM NaCl, 30 mM imidazole, pH 7.4, bound proteins were eluted with 20 mM Na_2_HPO_4_, 500 mM NaCl, 500 mM imidazole, pH 7.4 and the samples were dialyzed against PBS for 16 h at 4°C using SnakeSkin™ Dialysis Tubing 10K MWCO (Thermo Fisher Scientific).

### SDS-PAGE and immunoblot analysis

Total soluble protein (TSP) extracts were prepared by dissolving homogenized leaf material in 20 mM Na_2_HPO_4_, 500 mM NaCl, pH 7.4. Proteins separated by SDS-PAGE were either visualized by Coomassie Brilliant Blue staining or immunoblotting with anti-His (Thermo Fisher Scientific) or anti-RBD (Sino Biological) antibodies. For *in vitro* deglycosylation, purified RBD-215 or total soluble protein extracts were incubated with endoglycosidase H (EndoH) (New England Biolabs) or peptide-N-glycosidase F (PNGaseF) (New England Biolabs) according to the manufacturer’s instructions and subjected to SDS-PAGE and immunoblotting.

### MS analysis

Purified RBD-215 was S-alkylated with iodoacetamide and digested in-solution with endoproteinases LysC (Roche) and GluC (Promega). Glycopeptides were analysed using a Vanquish™ Neo UHPLC (Thermo Fisher Scientific) system coupled to an Orbitrap Exploris 480 mass spectrometer (Thermo Fisher Scientific). The possible N-glycopeptides were identified as sets of peaks consisting of the peptide moiety and the attached N-glycan varying in the number of HexNAc units, hexose, pentose and deoxyhexose residues. The theoretical masses of these glycopeptides were determined with an EXCEL spread sheet using the monoisotopic masses for amino acids and monosaccharides. The expected glycopeptides were manually detected and analysed with FreeStyle 1.8 program (Thermo Fisher Scientific). The deconvoluted, positively charged glycopeptides are shown in a mass range of 1900-4200 Da.

### ELISA

Plant-produced RBD-215 variants (6 nM) in PBS were coated onto NUNC Maxisorp 96 well plates (Thermo Fisher Scientific) overnight at 4°C. Plates were washed 3 times with PBS supplemented with 0.1% (v/v) Tween 20 (PBST) and subsequently blocked for 1 hour with 1% (w/v) BSA in PBST. Recombinant S309 (VIR7831) IgG1 antibody (a kind gift from Hugo Mouquet) (Planas et al., 2022) or plant-produced P5C3 IgG1 (Kallolimath et al., 2023) was diluted in PBST supplemented with 1% BSA (4-fold dilution series starting with 4 nM) and incubated for 2 hours. The plates were washed 3 times with PBST and incubated for 1.5 hours with anti-IgG (H+L) horseradish peroxidase conjugated antibody (Promega) diluted 1:5000 in PBST + 1% (w/v) BSA. After 3 washes, substrate solution (10 mM sodium acetate, pH 5 + 1:60 diluted TMB-stock solution (0.4% (w/v) tetramethylbenzidine (Fluka) in DMSO) + 1:300 diluted H_2_O_2_ (0.6% in H_2_O)) was applied (150 μL/well) and plates were incubated for 5-10 min with shaking. Reactions were stopped by the addition of 1 M sulfuric acid (25 μL/well) and absorbance was measured at 450 nm on a Tecan Sunrise Microplate (Tecan) reader using a reference wavelength of 620 nm. All samples were analysed at least twice with three technical replicates. EC50 values were calculated by non-linear regression of the blank-corrected data points based on a four-parametric log model with GraphPad Prism version 9.0 (Kallolimath et al., 2023).

### SPR

The surface plasmon resonance (SPR) experiments were performed using a Biacore T200 (Cytiva). All assays were performed with HBS-EP running buffer (Cytiva) at 25°C. To determine the binding kinetics between the RBD-215 variants (MW = ~24 kDa without glycans) and ACE2-Fc (MW = 189,8 kDa, produced in HEK293 cells) (Castilho et al., 2021), a CM5 chip was coated with 150 pg of Protein A via amine coupling in the active cell (5500 RU). The reference flow cell was left blank without Protein A. The ligand ACE2-Fc was immobilized non covalently at 2.63 nM. The analytes, RBD-215 variants, were injected over the two flow cells at a range of five concentrations (200, 80, 32, 12.8 and 5.12 nM) prepared by serial 2.5-fold dilutions, at a flow rate of 30 μL/min using a single-cycle kinetics program. Prior to SPR measurements protein concentration for each sample was verified by averaging 5 independent measurements taken with a NanoDrop spectrophotometer in the UV-mode (at 280 nm using sample-specific extinction coefficients). Running buffer was also injected using the same program for background subtraction. The chip was regenerated at each measurement with 10 mM glycine-HCl pH 1.7. The ligand ACE2-Fc was captured at every run. All data were fitted to a 1:1 binding model using Biacore T200 Evaluation Software 3.1. Measurements were repeated three times for RBD-215 variants and are reported with standard deviation. The sensorgrams were plotted using GraphPad Prism version 9.0.

### DSF

Protein stability measurements were carried out by differential scanning fluorimetry (DSF) using the CFX96 Real-Time PCR Detection System (Bio-Rad) with a final dilution of 1:500 of the SYPRO Orange dye (Molecular Probes). Fluorescence of a 25 μL sample (final concentration 0.4 mg/mL) in PBS was recorded from 10 - 95°C (0.5°C increments, 10 seconds hold per step) using the FRET channel. The thermograms, both the normalized relative fluorescence units (RFU) and the normalized derivative of relative fluorescence units (d(RFU)/dT) with respect to temperature (T) were recorded and compared. The peaks of the d(RFU)/dT-T thermogram are regarded as the melting temperatures (Tm) of the corresponding protein. Triplicate measurements were performed for each protein.

## Data availability statement

The datasets presented in this study can be found in online repositories. The names of the repository/repositories and accession number(s) can be found below: https://www.ebi.ac.uk/pride/archive/, PXD044462.

## Author contributions

VR: Conceptualization, Investigation, Visualization, Writing – review & editing. UV: Formal Analysis, Investigation, Writing – review & editing. JK-B: Formal Analysis, Investigation, Methodology, Writing – review & editing. OB: Formal Analysis, Investigation, Writing – review & editing. SK: Resources, Writing – review & editing. DM: Formal Analysis, Writing – review & editing. CG-G: Data curation, Investigation, Writing – review & editing. RS: Conceptualization, Data curation, Funding acquisition, Supervision, Writing – original draft.
