# Supplementary material for: Impact of mutations on the plant-based production of recombinant SARS-CoV-2 RBDs
Source: Front Plant Sci. 2023 Oct 6;14:1275228. doi: 10.3389/fpls.2023.1275228 (PMC10588190; doi:10.3389/fpls.2023.1275228)
Supplement: Supplementary file 1 [file DataSheet_1.pdf]

Supplementary Material

Impact of mutations on the plant-based production of recombinant SARS-CoV-2 RBDs

Valentina Ruocco[1], Ulrike Vavra[1], Julia König-Beihammer[1], Omayra C. Bolaños-Martínez[1], Somanath Kallolimath[1], Daniel Maresch[2], Clemens Grünwald-Gruber[2], Richard Strasser[1*]

[1]Department of Applied Genetics and Cell Biology, University of Natural Resources and Life Sciences, Vienna, Austria

[2]Core Facility Mass Spectrometry, University of Natural Resources and Life Sciences, Vienna, Austria

*Correspondence:

Richard Strasser

Email: richard.strasser@boku.ac.at

Keywords: antigen, glycoprotein, glycosylation, *Nicotiana benthamiana*, spike protein, vaccine, virus

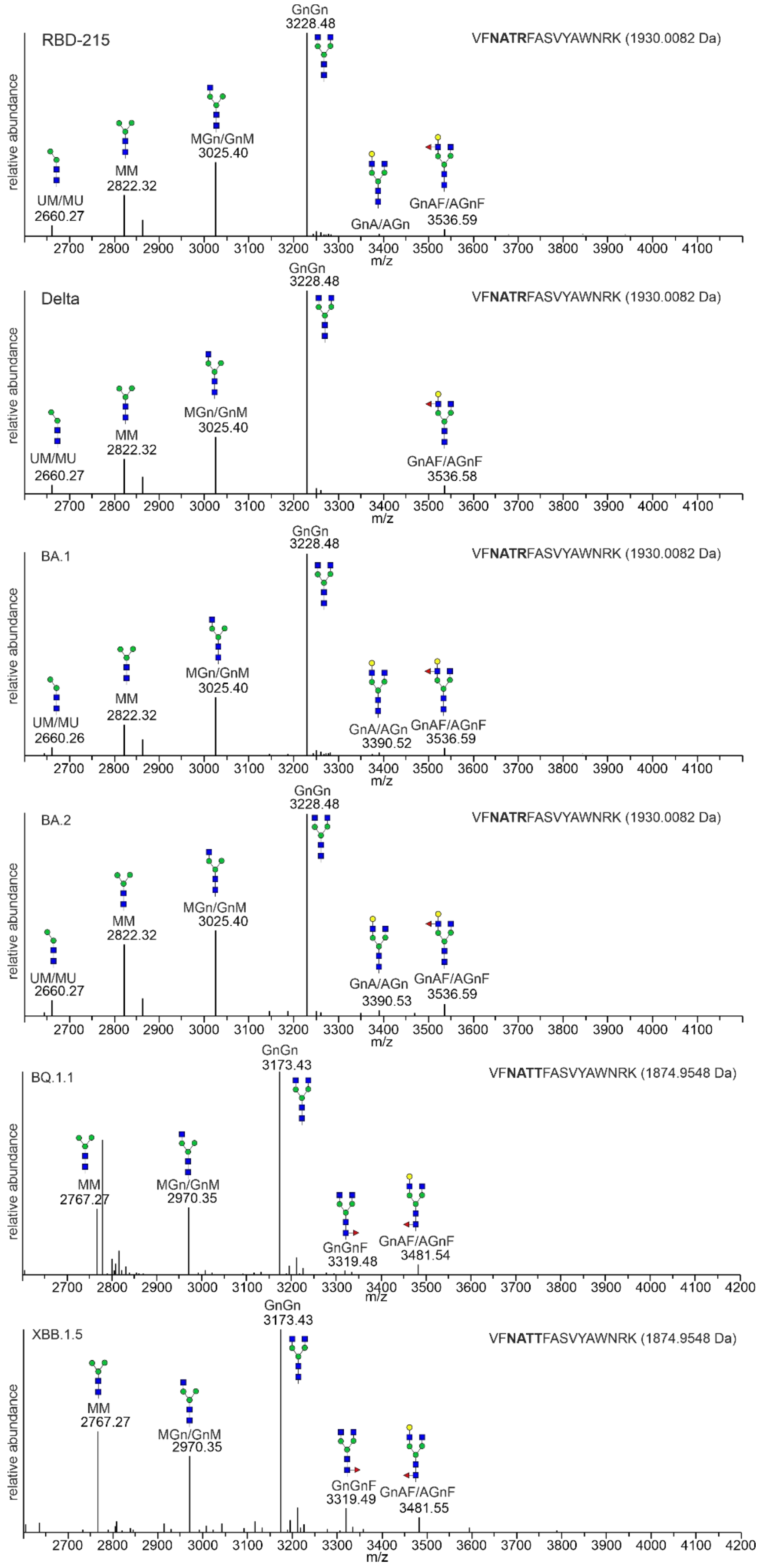

RBD-215
VFNATRFASVYAWNRK (1930.0082 Da)
GnGn 3228.48
MGn/GnM 3025.40
MM 2822.32
UM/MU 2660.27
GnA/AGn
GnAF/AGnF 3536.59
Delta
VFNATRFASVYAWNRK (1930.0082 Da)
GnGn 3228.48
MGn/GnM 3025.40
MM 2822.32
UM/MU 2660.27
GnAF/AGnF 3536.58
BA.1
VFNATRFASVYAWNRK (1930.0082 Da)
GnGn 3228.48
MGn/GnM 3025.40
MM 2822.32
UM/MU 2660.26
GnA/AGn 3390.52
GnAF/AGnF 3536.59
BA.2
VFNATRFASVYAWNRK (1930.0082 Da)
GnGn 3228.48
MGn/GnM 3025.40
MM 2822.32
UM/MU 2660.27
GnA/AGn 3390.53
GnAF/AGnF 3536.59
BQ.1.1
VFNATTFASVYAWNRK (1874.9548 Da)
GnGn 3173.43
MM 2767.27
MGn/GnM 2970.35
GnGnF 3319.48
GnAF/AGnF 3481.54
XBB.1.5
VFNATTFASVYAWNRK (1874.9548 Da)
GnGn 3173.43
MM 2767.27
MGn/GnM 2970.35
GnGnF 3319.49
GnAF/AGnF 3481.55
relative abundance
m/z

Figure S1. Representative MS-spectra of the RBD-215 glycopeptide harbouring N-glycosylation site N343 of the SARS-CoV-2 spike protein. In the shown spectra, the peaks at 3228.48 and 3173.43, respectively, are assigned to the complex N-glycan GnGn. Additional complex (MGn/GnM, GnGnF, GnA/AGn, GnAF/AGnF) and truncated (MM, UM/MU) N-glycans are indicated (nomenclature according to the ProGlycAn system: www.proglycan.com). The illustration (blue squares, GlcNAc; green cycles, mannose; red triangle, fucose; yellow cycles, galactose) indicates the most likely N-glycan corresponding to the mass (other isoforms are possible).

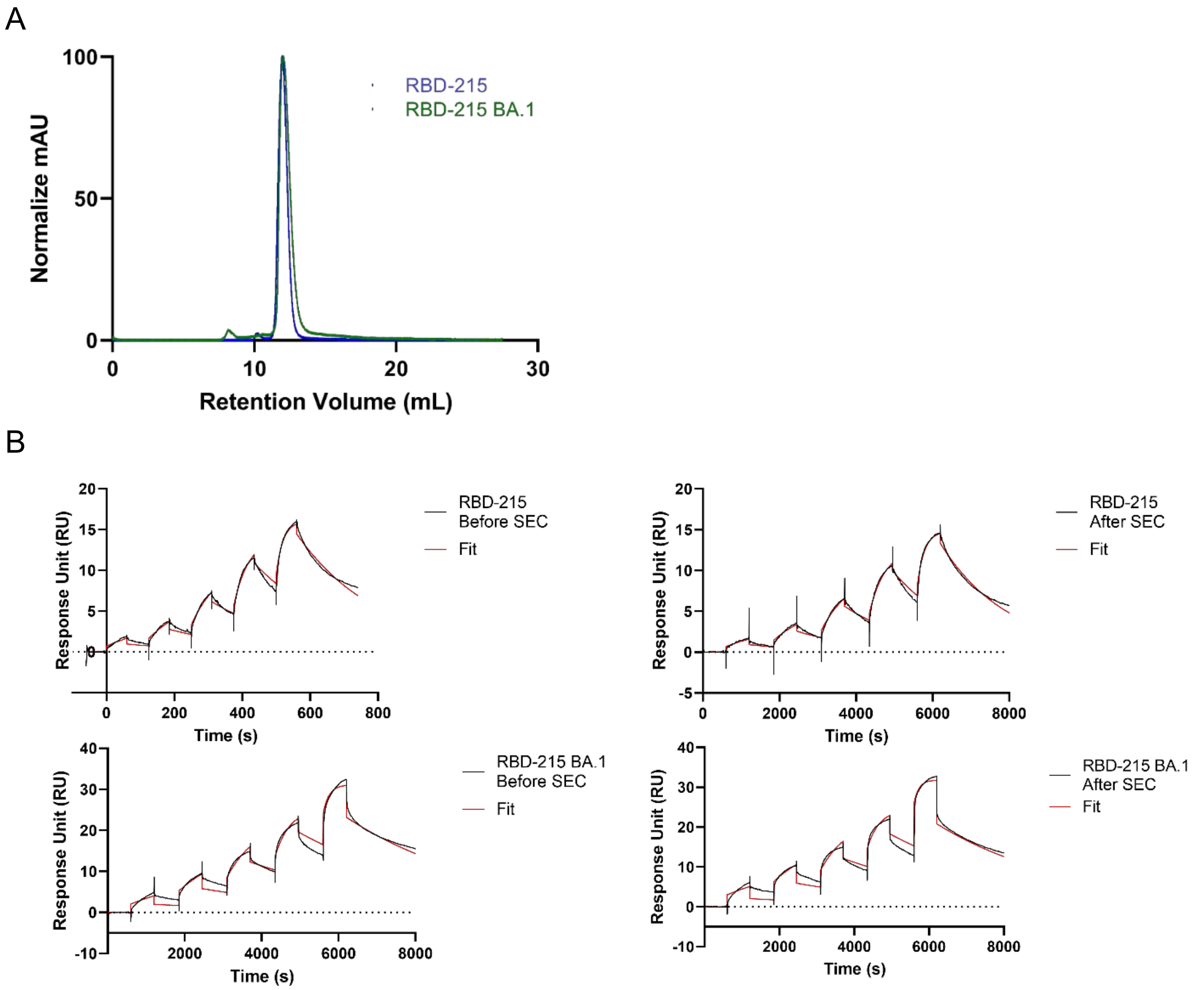

	ka (1/Ms)	kd (1/s)	K_D (nM)
RBD-215 Before SEC	2.3×10^5	41×10^{-4}	18.3
RBD-215 After SEC	2.4×10^5	57×10^{-4}	23.5
RBD-215 BA.1 Before SEC	3.0×10^5	27×10^{-4}	8.9
RBD-215 BA.1 After SEC	3.6×10^5	28×10^{-4}	7.8

Figure S2. Comparison of SEC and non-SEC purified RBD-215 and BA.1. (**A**) SEC chromatograms showing the amounts of monomers (major peak), dimers or oligomers. (**B**) SPR sensorgrams and kinetic parameters. Independent batches of RBD-215 and BA.1 were purified and analysed. Therefore, the K_D values are slightly different from the ones shown in Figure 5 and Table S2.

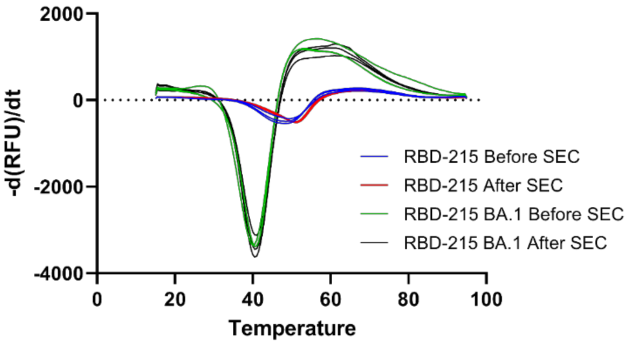

	Tm (°C)
RBD-215 Before SEC	48,8 ± 0,6
RBD-215 After SEC	50,8 ± 0,3
RBD-215 BA.1 Before SEC	40,3 ± 0,3
RBD-215 BA.1 After SEC	40,6 ± 0,3

Figure S3. DSF profiles and Tm values of SEC and non-SEC-purified RBD-215 and BA.1. Independent batches of RBD-215 and BA.1 were purified and analysed. Therefore, the Tm values are slightly different from the ones shown in Figure 6.

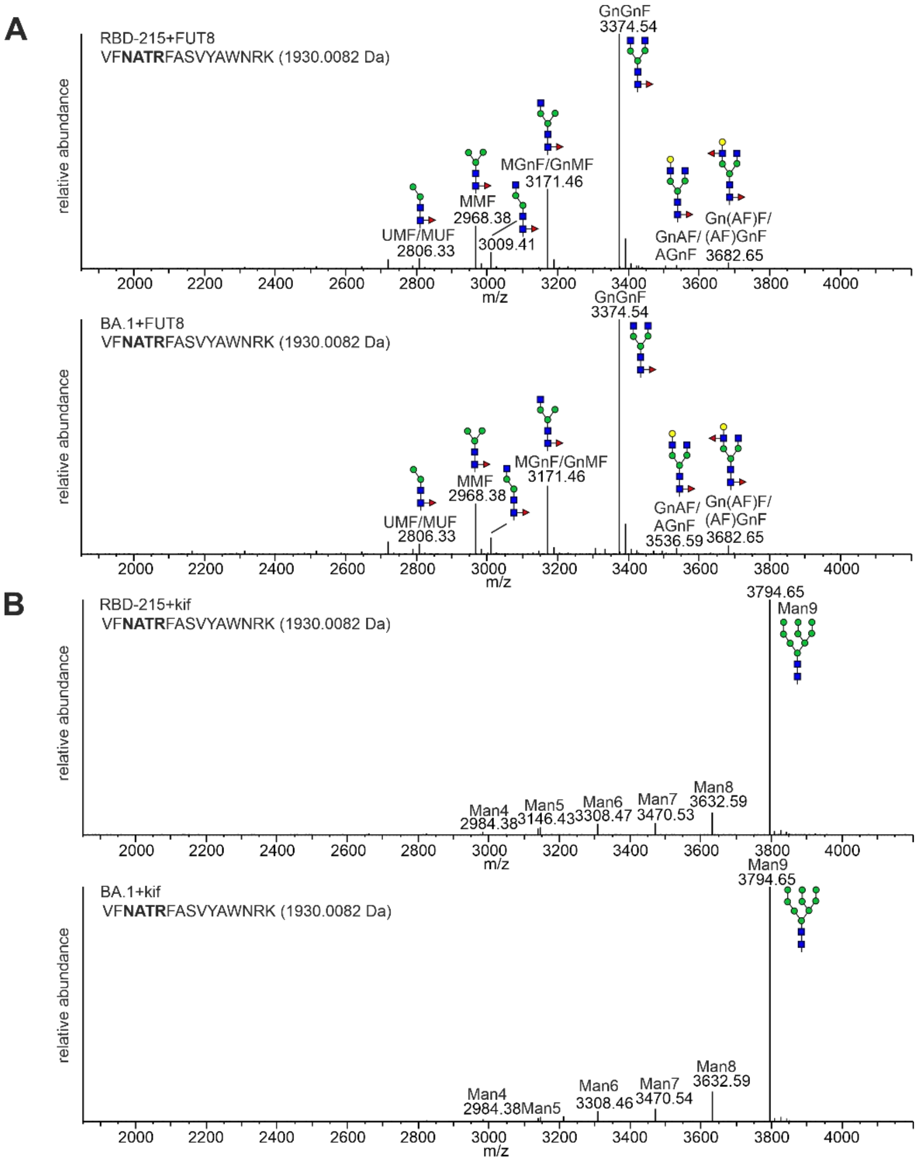

Figure S4. Representative MS-spectra of the RBD-215 glycopeptide harbouring N-glycosylation site N343 of the SARS-CoV-2 spike protein. In the shown spectra in (**A**), the peak at 3374.54 is assigned to the complex N-glycan GnGnF (blue squares, GlcNAc; green cycles, mannose; red triangle, fucose; yellow circle, galactose; nomenclature according to the ProGlycAn system: www.proglycan.com). In the spectra in (**B**), the major peak at 3794.65 is assigned to the oligomannosidic N-glycan Man9 ($Man_9GlcNAc_2$). Other mannosidic N-glycans are also indicated.

Table S1. Root Mean Square Deviation (RMSD) of the pruned atom pairs and the RMSD across all pairs corresponding to the visualization of structural similarities and dissimilarities as shown in Fig. 1D. The alignment process aligns the RBD-215 structures of all variants to the ancestral RBD-215, and the resultant superimposed ribbon diagrams effectively emphasize areas of both structural similarity and dissimilarity.

	RMSD, *Å* Pruned atom pairs	RMSD, *Å* All pairs
RBD-215 Delta	0.511	1.945
RBD-215 BA.1	0.452	7.702
RBD-215 BA.2	0.621	9.064
RBD-215 BQ.1.1	0.678	5.220
RBD-215 XBB.1.5	0.796	3.439
RBD-215 BA.2.75	0.650	8.535
RBD-215 BA.4/5	0.569	3.382

Table S2. Binding kinetics of RBD-215 variants to ACE2-Fc as measured by SPR. Values (mean ±SD, n = 3) for association rate (ka), dissociation rate (kd), and kinetic dissociation constant (K_D) are shown.

	ka (1/Ms)	kd (1/s)	K_D (nM)
RBD-215	$3.7 \times 10^5 \pm 0.5 \times 10^5$	$55 \times 10^{-4} \pm 2 \times 10^{-4}$	14.8 ± 0.7
RBD-215 Delta	$5.9 \times 10^5 \pm 0.8 \times 10^5$	$31 \times 10^{-4} \pm 4 \times 10^{-4}$	5.2 ± 0.1
RBD-215 BA.1	$4.3 \times 10^5 \pm 0.5 \times 10^5$	$30 \times 10^{-4} \pm 3 \times 10^{-4}$	6.9 ± 0.1
RBD-215 BA.2	$6.4 \times 10^5 \pm 0.9 \times 10^5$	$32 \times 10^{-4} \pm 3 \times 10^{-4}$	5.0 ± 0.2
RBD-215 BQ.1.1	$4.0 \times 10^5 \pm 0.4 \times 10^5$	$12 \times 10^{-4} \pm 2 \times 10^{-4}$	2.9 ± 0.2
RBD-215 XBB.1.5	$5.3 \times 10^5 \pm 0.5 \times 10^5$	$12 \times 10^{-4} \pm 2 \times 10^{-4}$	2.2 ± 0.1

	ka (1/Ms)	kd (1/s)	K_D (nM)
RBD-215 + kif	$5.2 \times 10^5 \pm 0.3 \times 10^5$	$69 \times 10^{-4} \pm 4 \times 10^{-4}$	13.1 ± 0.6
RBD-215 BA.1 + kif	$2.7 \times 10^5 \pm 0.7 \times 10^5$	$22 \times 10^{-4} \pm 3 \times 10^{-4}$	8.2 ± 0.2
RBD-215 + FUT8	$4.2 \times 10^5 \pm 0.3 \times 10^5$	$44 \times 10^{-4} \pm 5 \times 10^{-4}$	10.5 ± 0.5
RBD-215 BA.1 + FUT8	$5.9 \times 10^5 \pm 0.5 \times 10^5$	$26 \times 10^{-4} \pm 2 \times 10^{-4}$	4.4 ± 0.1
